# Peri-Urbanism in Globalizing India: A Study of Pollution, Health and Community Awareness

**DOI:** 10.3390/ijerph14090980

**Published:** 2017-08-30

**Authors:** Linda Waldman, Ramila Bisht, Rajashree Saharia, Abhinav Kapoor, Bushra Rizvi, Yasir Hamid, Meghana Arora, Ima Chopra, Kumud T. Sawansi, Ritu Priya, Fiona Marshall

**Affiliations:** 1 Institute of Development Studies, University of Sussex, Brighton BN1 9RE, UK; 2 Centre of Social Medicine and Community Health, Jawaharlal Nehru University, New Delhi 110067, India; ramila.bisht@gmail.com (R.B.); rajashree.raj8@gmail.com (R.S.); Kapoor.abhinav123@gmail.com (A.K.); ushirizvi123@gmail.com (B.R.); meghana.arora@gmail.com (M.A.); imachopra@gmail.com (I.C.); kumudteresa@gmail.com (K.T.S.); ritu_priya_jnu@yahoo.com (R.P.); 3 Department of Psychology, University of Kashmir, Hazratbal, Srinagar, Jammu and Kashmir 190006, India; yasu8001@gmail.com; 4 Science Policy Research Unit (SPRU) and STEPS Centre, University of Sussex, Brighton BN1 9RH, UK; f.marshall@sussex.ac.uk

**Keywords:** peri-urban, agriculture, water pollution, industrial pollution, health, collective action, urbanization

## Abstract

This paper examines the intersection between environmental pollution and people’s acknowledgements of, and responses to, health issues in Karhera, a former agricultural village situated between the rapidly expanding cities of New Delhi (India’s capital) and Ghaziabad (an industrial district in Uttar Pradesh). A relational place-based view is integrated with an interpretive approach, highlighting the significance of place, people’s emic experiences, and the creation of meaning through social interactions. Research included surveying 1788 households, in-depth interviews, participatory mapping exercises, and a review of media articles on environment, pollution, and health. Karhera experiences both domestic pollution, through the use of domestic waste water, or *gandapani*, for vegetable irrigation, and industrial pollution through factories’ emissions into both the air and water. The paper shows that there is no uniform articulation of any environment/health threats associated with *gandapani*. Some people take preventative actions to avoid exposure while others do not acknowledge health implications. By contrast, industrial pollution is widely noted and frequently commented upon, but little collective action addresses this. The paper explores how the characteristics of Karhera, its heterogeneous population, diverse forms of environmental pollution, and broader governance processes, limit the potential for citizen action against pollution.

## 1. Introduction

This paper examines the intersection between environmental pollution and people’s acknowledgement of health concerns in peri-urban India. These peri-urban spaces have been “crying out for attention” [1] and are places where neoliberal policies, real-estate booms, land speculation, information technology advances, and the relocation of industrial waste have “transformed the pace of development” [2]. They are the consequence of urban expansion and a visible manifestation of urban socio-spatial inequalities. One such area, explored in this paper, is situated between the rapidly expanding cities of New Delhi (India’s capital) and Ghaziabad (an industrial district in Uttar Pradesh), where urban growth has created a peri-urban interface; “a territory between” rural and urban livelihoods, activities, and services [3]. Peri-urban spaces are generally characterized by a predominance of poor and disadvantaged residents; a lack of services, infrastructure and facilities; degraded natural resource systems [4]; and, industrial hazards [1,2,5]. This research focuses on Karhera, a peri-urban village sandwiched between Delhi and Ghaziabad, which was, until about two decades ago, predominantly agricultural. Despite being surrounded by urban growth, some of Karhera’s residents have continued to practice agriculture. This emphasis on agriculture forms an important component of Karhera and sets it apart from the rapidly-urbanizing spaces around it. However, the nature and scale of this agriculture has changed considerably. Cereal crops (maize, wheat, rice and sorghum) and vegetables have been replaced by spinach, grown to take advantage of the urban demand for fresh vegetables. Urbanization has also affected the natural environment and the resources used for agriculture, with less available land for cropping and fewer spaces to keep livestock. Other environmental resources—such as water—have become polluted and degraded, making it hard to continue farming. Not all is negative however, and these changes have been accompanied by new farming opportunities. Karhera is thus an ideal context in which to explore the significance of peri-urban places in relation to “dynamics, diversity and complexity” of pollution and health [6,7]. Such places have particular place-based characteristics and face significant sustainability challenges [8,9]. Karhera, like India’s other peri-urban places, is also the physical manifestation of the globalization processes—created and molded by—regional agglomeration, liberalization, urbanization, global economic integration, and “re-structuring for globalized systems of production and consumption” [10,11].

Considerable attention has focused on how communities identify pollutants and toxins in their localities, and on how their mobilization can and does effect change. Much of this has emphasized participatory processes, in particular the collective actions undertaken in the form of popular epidemiology, often in conjunction with sympathetic researchers to co-produce scientific evidence of pollution and ensure engagements with policy makers [12,13,14,15,16]. While collective mobilization is seen as a viable means of challenging vested interests and of forcing government and policy actors to pay attention to environmental pollution and health threats [17], collective activism and citizen science research has not always guaranteed such emancipated outcomes [12]. Until recently, however, little attention has been paid to contexts where citizen science alliances do not occur, and where community residents fail to publically acknowledge—and do not collectively act upon—the intersections between environmental pollution and health. This recent research has explored the factors which constrain mobilization, and in doing so, have shown that this is not simply a result of the well-known diversionary tactics used by the state and political and economic elites (such as media control, limiting citizen participation, emphasizing the economic benefits; discrediting scientific evidence of harm and health, casting blame on potential sources of contamination, and bullying, ostracizing, and discrediting activists) to forestall resistance [18]. Adams and Shriver [19] show, for example, how protests against coal mining in Czechoslovakia struggle to direct their activism as economic and political flux create a situation where the targets are vague and ambiguous. Auyero and Swinstun [20] explore the “slow violence” perpetrated by petrochemical companies in an Argentinian Shantytown, where the environmental damage is neither dramatic nor visible. Here the slow accretion of toxins results in long-term habituation which in turn has meant that, even once the extent of the damage was known, residents remained uncertain and conflicted about their exposure to contamination and the associated risk. This research has also recognized that citizens may welcome the economic opportunities that produce environment degradation and corresponding health risks because of their poverty, economic dependence, and marginalization [18,21,22]. Underlying all of this work is an emphasis on place and economic context as shaping people’s responses to environmental pollution and health threats, showing that the “relational interplay between place characteristics and their meaning-making for health is often contingent and contested” [7]. Whereas, environmental and health hazards in peri-urban areas have been thoroughly documented [23,24], this paper adds to an emerging body of work which examines people’s conflicted identification of health and environment threats in peri-urban areas in the global south, and the place-based factors which shape the potential for collective action against environmental pollution.

This article addresses this knowledge gap by exploring the peri-urban area of Karhera where, despite emerging environment and health threats associated with urbanization, there is little evidence of citizen mobilization tackling these issues. We investigate why this might be by constructing a descriptive demographic and social profile of area and its residents, and by exploring their perceptions and feelings regarding the intersection of environmental pollution and health in their rapidly changing peri-urban environment.

After outlining methods used in this research, this article goes on to present findings including the contextual and demographic profile of the area, and how it has changed over time as a result of urbanization. Highlighted are changes in the socio-economic composition of the area, shifts in land and water use/availability, and associated livelihood strategies, as well as increasing levels of apparent environmental pollution, growing potential health risks, and the perceptions of residents around these issues.

This is then followed by a more in-depth discussion section which synthesizes these multiple shifts and the perceptions of residents to explore how these dynamics interact with each other in complex ways that ultimately undermine the potential for collective action. By making these underlying dynamics visible, this article provides important insights into the challenges faced by citizens and civil society groups who seek to build collective action movements against pollution and health risks in peri-urban areas.

## 2. Materials and Methods

In this paper, we integrate a relational view of place—which sees place as “having physical and social characteristics... [which] are shaped by and given meaning through their interactions with politics and institutions, with one another and, most importantly, with the people living in a place” [7]—with an interpretive approach, which seeks to integrate people’s views with “an analysis of cultural phenomena, social conditions and structural constraints” [25]. We focus on people’s diverse interpretations of facts, their emic experiences, on how they create meaning through social interactions; on how relationships and social dynamics have changed over time, on the effects of formal policies, and on political processes and power relations. Fieldwork was undertaken in Karhera, Ghaziabad District between August 2014 and May 2015, using a variety of fieldwork methods which sought to capture multiple ways of portraying and comprehending the place, Karhera [7]. This included surveying 1788 of the 2042 households in September 2014. The survey asked about household composition, caste, primary and secondary sources of livelihood, home ownership, perceptions of health and hospitalization (over the past 5 years), and land ownership. The survey was conducted by the authors of this paper, based at the Centre of Social Medicine and Community Health, Jawaharlal Nehru University. Researchers aimed to survey every household in Karhera, but 152 households were unwilling to be interviewed, and in 102 households no-one was home and doors were locked. Given that 88% of households ultimately participated in the survey, it is reasonable that the data is representative of the community as a whole. The researchers asked questions and completed the answers on paper survey instruments. The data was processed with SPSS version 22.0 (IBM Corporation, New York, NY, USA) and used to identify emergent themes around environmental degradation, pollution, health, and emergent risk.

Qualitative research methods, undertaken between October 2014 and May 2015, provided a detailed understanding of agricultural livelihoods in relation to changing circumstances and urbanization. Twenty in-depth semi-structured interviews were undertaken, 10 with men and 10 with women who were identified from the survey. The following criteria informed our selection: involved in agriculture (transporting, buying or selling produce, growing crops, working as laborers, sharecroppers, or leasing agricultural land), migrant household men and women or men and women who were original inhabitants and were willing to participate in the research. Informants came from different castes and socio-economic groups. These discussions usually lasted about an hour, and interviews took place in locations that were mutually convenient such as participants’ homes or fields. These face-to-face interviews were conducted by the researchers in Hindi, recorded, translated and transcribed. The interviews examined agricultural-based livelihoods, people’s use of natural resources (water, firewood etc.), and personal experiences of urbanization, poverty, and community relations. We then undertook four participatory mapping sessions with (a) men involved in agriculture; (b) women involved in agriculture; (c) men who were actively farming or marketing spinach; and (d) women associated with buffalo rearing and/or spinach. Participants were recruited in ways customary to anthropological research practice: through formal and informal interaction with the researchers, who lived in the community for periods of the research. These sessions, which lasted half a day and took place in a local Hindu temple, brought together small groups of people (between 5 and 12) associated with the dominant crop (spinach) and animal husbandry to collectively hand-draw maps of the Karhera they had known 20 years ago. As a part of the participatory mapping sessions, participants reflected on changing agricultural livelihoods; new uses of space and resources; and, the implications of these on labor practices, water availability, and gender relations. They also discussed topics such as food availability, distribution and exchange; food preferences; the nutritional and social values associated with agricultural crops; changing political relations associated with access to land and water; and, poverty and health. The interviews and participatory mapping sessions complemented the survey by providing further insights into the relationships between agriculture, livelihoods, and people’s collective responses to social and environmental change and by facilitating triangulation of patterns and trends.

Finally, a review of media articles on environment, pollution, and health in the Trans-Hindon, between 2005 and 2015, was undertaken. This was initiated by searching the Centre for Science and Environment (CSE) website. The CSE website has an India Environment Portal (http://www.indiaenvironmentportal.org.in/) which archives articles from leading newspapers, books, magazines, etc. related to issues of environmental concern. The CSE’s magazine, “Down to Earth”, was searched for significant articles related to Hindon river and Trans-Hindon region and two English daily newspapers website (The Hindustan Times and The Hindu) and two regional-language daily newspapers website (Jagran and Amarujala) were selected because of their popularity and readership in the region. The search terms identified for this stemmed from the qualitative research and were: agriculture, fertilizer, pesticides, health risks, diseases, cancer, pollution, waste water, effluents, industrial waste, poverty, and urbanization. Special attention was focused on articles that linked environment, pollution and health with political mobilization. This exercise allowed us to see what types of pollution and, health issues were being taken up by local newspapers and which may reflect, or influence, residents’ levels of concern.

Standard social science ethical procedures were followed, including adhering to the principles of informed consent and confidentiality. Pseudonyms are therefore used in this paper. Participants were clearly informed that they could withdraw at any time without facing negative repercussions for doing so. Ethical approval was received from the University of Sussex (ER/PLW20/1).

Of final methodological consideration are the strengths and limitations of this study. While this research offers unique insights into Karhera’s residents’ perceptions of environmental pollution and health risks in their changing locality, it cannot provide hard evidence on the links between pollution and resulting health problems as scientific investigations were not undertaken. That said, the strength of this paper lies in its exploration of local perceptions of pollution and health risks in contexts where these are not highly evident, and assessing the implications of this for potential future mobilization. Another limitation of this study is that it was not designed for replicability. This is not unusual in relational, place-based research where emphasis is on situating people’s personal accounts within socio-economic and political broader contexts associated with a very specific place.

## 3. Findings

### 3.1. Ghaziabad and Karhera: Context and Demographics

Ghaziabad District consists of four *Tehsils* (divisions), the largest, and most densely populated of which is Ghaziabad *Tehsil*. It includes a diversity of urban and peri-urban settlements, with people variously accommodated in villages, unauthorized colonies, slums, and middle-class colonies. This area has been enormously affected by the transformations in nearby Delhi—for example, through the loss of farmland to urban development and through the relocation of polluting industries from Delhi to Ghaziabad [26,27]. The population in Ghaziabad has grown as people are attracted by its urbanizing nature, including rural migrants looking for work and urban populations relocating because of cheaper housing and improved commuting possibilities.

Karhera is a former agricultural village situated within the Ghaziabad Municipal Corporation, an administrative area of Ghaziabad *Tehsil*. In 1987 this area was converted into a *Nagar Parishad*, a designation indicating its urban status. Karhera is bounded, on one side of the village, with a line of industries and production units. The other side of Karhera is bounded by the Hindon River.

In the 2014 survey undertaken as a part of this research, almost half of the people surveyed were original inhabitants who lived in Karhera when it was an agricultural village (44%), while the remainder were migrants (56%), attracted by the industries and the potential for work in nearby cities. Kahera thus has a heterogeneous population, which includes people of different castes, religions, and geographic origins. The 2014 survey shows that three quarters of the original inhabitants (75%) are upper-caste (primarily *Rajput*), and the remaining quarter is of lower-caste origin (primarily *Dalit*).

During participatory mapping exercises, Karhera’s original residents reported that, 20 years ago, they had relied on agriculture as a primary source of livelihood. By 2014, our survey showed that only 24% of karhera’s households (16% of original households and 8% of migrant households) still cited agriculture as their primary source of income. Agriculture, has, with increasing urbanization, become a secondary occupation and increasingly feminized. Animal husbandry too has decreased, and is now primarily for subsistence purposes. More than a third of both original and migrant households, 41% in the case of original inhabitants and 37% of migrants, depend on desk-based private sector employment such as teaching, insurance, working in call centers, and as estate dealers. A further 18% of original and 10% of migrant households have businesses as their primary source of income. These include repair and grocery shops, transport businesses, factories, and garages. Only a small percentage of original inhabitants (6%) and no migrants held government posts or were former-government employees. Only 15% of the original households, as compared to 30% of migrant households, relied on manual labor as their primary livelihood (drivers, factory workers, and mechanics). The majority of these manual laborers are lower-caste.

### 3.2. Transformations and Pollution in Karhera

Urbanization has involved several major transitions in terms of how busy and built up the area is, changes to the water supply and reductions in land availability; in conjunction with environmental degradation. Karhera, once a quiet rural village, is now a bustling area. New roads connect Ghaziabad to Delhi, traffic is constant and accompanied by noise, people, and pollution. New buildings, shopping malls, and urban activities now characterize the area [3,28].

Water has become scarcer. Large amounts of water are consumed by the industrial sector and by the government of Ghaziabad, which has installed submersible water pumps in peri-urban locations in order to supply water to the new urban establishments, including high-rise apartments and malls. The result has been a lowering of the water table. Areas such as Karhera have been affected by these urbanization processes, and their tubewells no longer provide adequate sources of water [3,26]. Water shortages have also reduced the amount of land suitable for cultivation. For example, much of the Hindon River bank is no longer suitable for cultivation. Agricultural land has also been diverted to urban use. This includes industrial clusters, infrastructure construction, new roads, real-estate development, and urban leisure activities. More specifically, the government has acquisitioned—over the years—Karhera’s rural land: 42 acres in the 1960s for the Hindon Air Force base and the creation of Loni Industrial area; 104 acres for the 1987 Vasudhara Vikas Awas scheme and new urban settlements; and, land for the Ghaziabad Master Plan which came into force in 2005 [28]. In 2014, when a “City Forest” was created to meet the leisure and greening demands of an urban middle-class population, and a flyover and power station were built. Although some farmers reported receiving limited compensation for these land acquisitions, during interviews and mapping sessions, Karhera’s residents emphasized that land had been taken from them. They have also, in the early 1990s, sold land to outsiders who built new residences in the “new Karhera colony”. At the time of the research, the proposed development of the metro line led Karhera’s land-holders to believe that they would soon lose more land.

### 3.3. Agriculture in a Rapidly Urbanizing Context

In 2014, almost half of Karhera’s households (42%) still relied on agriculture to make some contribution towards their livelihoods. For just under a quarter of households (24%), it was their primary source of livelihood and it provided a secondary income for nearly a fifth of households (18%). There have nonetheless been significant shifts from predominantly cereal-based farming to intensive, small-scale spinach farming. This contrasts to Karhera’s previous status, a primary agricultural area with a wide range of staple and vegetable crops, best known for its wheat and carrots.

Irrigated agriculture occurs on fields located about 4 km from Karhera’s residential area. These fields are close to the Hindon, and have traditionally been irrigated by water from the river and wells. Over time, as the water table dropped, farmers were compensated by using borewells. These in turn have dried up and those farmers who can afford to, have installed water pump submersibles. When talking about these fields, the villagers refer to crops grown in “clean water”. As shown above, however, both the Hindon and the ground water is highly polluted by industrial contaminants.

A collective irrigation system, designed and managed by Karhera’s residents and the *Panchayat* (the local form of village governance prior to Karhera becoming an urban ward), irrigates fields located closer to Karhera using domestic waste water (and where feasible tubewell water). Karhera’s villagers decided upon this irrigation system about 25 years ago when domestic wells were becoming increasingly saline/polluted, and other traditional water sources (community ponds or *jhora*) were filled-in to make way for new roads. Residents also installed submersibles to ensure their domestic water supply. These factors, in conjunction with piped water and new urban behaviors (daily washing) led to large quantities of domestic wastewater or *gandapani* (literally, dirty water). As submersibles were expensive and water precious, drains were built to direct *gandapani* (domestic wastewater) to irrigate these fields.

Whereas, people had previously grown a wide variety of crops, including wheat, rice, carrot, turnip, bottle gourd, sorghum, and fodder, using *gandapani* facilitated green leafy vegetable growing in response to urban market demands. Spinach soon became “the crop”. It thrived well in domestic waste, had a short production cycle, was in high demand, and was not liked by wild animals. As one resident explained during an interview: “It so happened that because of the shrinking of the forests, wild pigs and *NielGai* (antelope) started to destroy our standing crops”.

The wastewater used in the irrigation system was, at first, only from cooking and bathing. However, as submersibles were installed in the village, even more wastewater became available. This eased the villagers’ needs for domestic water and meant that women could wash clothing at home, rather having to go to the river or a stream. This also meant that flush toilets became more common, and as more and more migrants settled in Karhera, so the amounts of wastewater increased. In addition, once Karhera became an urban ward, the village *Panchayat* disintegrated. As a consequence, the drains were no longer maintained and *gandapani* came to contain fecal matter. Spinach thrives in this polluted water, growing very quickly and providing, all-year-round, a harvest every 20–30 days. A group of *Rajput* (upper-caste) elderly women discussed the subsequent prioritization of spinach farming in a participatory mapping exercise:

Carrot and wheat will take a minimum of 4 months to mature. But in the same time spinach grows throughout year and it takes hardly takes one month, now see this is one kiyari (plant bed), and in this kiyari the spinach is matured now. We will cut this and at the same time in the very next kiyari we sow another spinach. So it’s easy and it will work and carry on like this. It does not need much physical work, there is no need of plough the field, just give water, put the seed in or spread the seeds into the field. That’s all it needs.

During participatory sessions, residents explained that spinach farming is lucrative and has brought financial stability to those families engaged in cultivation. It has also enabled women to achieve financial independence. For example, a widow named Shanti Rani was one of the first women in Karhera to take up, and to survive exclusively on spinach cultivation. As other women, discussing Shanti explained: “Yes, we are making good money. Look she is growing spinach by herself; she cuts the harvest and sells in the market. So the money remains with her”.

In Karhera, spinach grown in *gandapani* is preferred to that grown in “clean water” because the use of wastewater reduces production costs (water is free and less fertilizer is required). In addition, there is no differentiation in sale price, and some men, in participatory mapping sessions, suggested that *gandapani* spinach is more marketable: “The spinach which is grown in unclean water sells faster in the market because of its shine. It shines because it is getting pure and natural dung so this is the difference”. Mother Dairy is the only buyer that specifies that crops must be irrigated in “clean water” crops. Created as a government subsidiary, this private company buys and sells agricultural produce. Vegetables and fruit are dealt with through Safal, which aims to establish a “direct link between growers and consumers” in order to provide fresh, healthy produce. In the Delhi metropolitan region, Safal is synonymous with “quality, trust and value”. However, Safal/Mother Dairy does not pay more for this spinach.

Arable land supplied with *gandapani* is highly desirable because of the access to free water; proximity to the village so less time is spent walking and transporting equipment and produce; and, because of the frequent and high spinach yields. Moreover, “clean water” fields require infrastructure to ensure constant irrigation. The tubewells and borings previously managed by that farmers no longer provide water because of the lowering of the groundwater table. The installation of submersibles is expensive and the benefits uncertain. As Suraj explains, “they also need to spend on generator to run the submersible and tractor to till the land. It means only those farmers who can spend around 5 *lakhs* (or 500,000 rupees) can cultivate”.

Take for example, the following two cases, both derived from the in-depth interviews:

Jayawati and her husband’s tubewells failed when the government submersible, which provides water to middle-class colonies in Ghaziabad, was installed. Previously it was possible to access ground water at 30 feet, but now, Jayawati says, it is below 250 feet. “Earlier we have boring in our field, but it does not work now”. They decided to install a submersible. “I cannot allow my children to starve, so I have taken a loan of 2 *lakh* rupees and have installed the submersible in the field”.

Sushil’s land is located near the foothills where there is no connectivity to the *gandapani* drainage system. The tubewell on his land failed due to the government submersible that has been installed alongside it. This has meant that he has to buy water at the rate of 350 rupees per hour. For him, agriculture has become very costly. After all the expenses on water and fertilizer; he makes only a small profit or, in his words, he “is hardly left with some money”.

As a consequence, some land owners allow their lands to lie barren as they cannot afford to farm. Others have, for the same reason, decided to sell. As one farmer explained, “Here the price of land is very high i.e., 50 thousand per *gaj* [square yard]. So, the farmers prefer to sell their lands, than starving”. *Gandapani* spinach farming is, however, thriving.

### 3.4. Environmental Pollution

Despite these pressures on land and water, almost half of Karhera’s population (42%) is involved in farming in one way or the other. This means that these people are intimately connected with the environment on a daily basis. They are the ones most affected by—and best placed to recognize—environmental pollution and degradation. They are the ones most likely to experience any health consequences because of their close contact with the water, air, and soil. In Karhera, pollution concerns primarily the water (described above) and the air.

As is the case in many of India’s peri-urban villages, Karhera’s residents are aware of industrial contamination. Industries are known to be pumping untreated water and effluents into the Hindon. This contamination has percolated the soil, making ground water unsuitable for human consumption. Industries and factories have also reportedly pumped toxic water directly into the water table. These forms of pollution are well recognized by Karhera’s residents, who all stressed the poor quality of water. They complain that this has polluted the river, which had previously been a significant source of water. One such example, shared in an interview, comes from Umesh, who has lived in Karhera all his life:

For the villagers Hindon happens to be a very good river. We used to drink water from Hindon. We used to take bath also. Sometimes while working in the fields we used to even drink water. The water of Hindon used to be so clean that you can easily see any coin falling there. However, with the coming of the industries, the river water started to become dirty. The drains of the cities were connected to Hindon; the drains of the factories also were connected. Till what level would the river bear this pollution?

A second example comes from Harish Singh, a retired veterinarian and now farmer from Karhera: “From the time when the factories started to drain out the contaminated water, from then onwards the Hindon River started to get polluted”. Nowadays, the water from the Hindon is, as villagers say, “black” from the factory drains, and is no longer used for irrigation or drinking. This polluted water has been linked to a range of diseases. As one upper-caste male vendor explained during the participatory mapping: “Look, some survey revealed that the Hindon River is causing cancer. There are cases of cancer in the village. Many people have been affected. The reason has been the [contamination of the] drinking water”. This articulation of environmental health threats is echoed by Jamuna Devi, also from Karhera. She said “all the health problems such as cancer, high blood pressure, and joint pains among the people of young age are caused by the water”.

Using sewage water for crop irrigation can also have negative consequences. Srinivasan and Reddy [29] argue that it can lead to increased levels of morbidity, and there is scientific evidence that heavy metal contamination in crops can stem from wastewater vegetable production, with spinach and other leafy vegetables being particularly prone to heavy metal uptake [30,31,32]. Growing spinach also requires significant amounts of time spent handling the crops and being exposed to the polluted water. This exposure can, after a few years, result in a range of ill-defined symptoms such as headaches, skin diseases, fever, stomach ailments, and diarrhea. Microbial infections (including pathogenic viruses, bacteria, and protozoa) may also be transmitted in this water. In the peri-urban areas of Hyderabad City, poor water quality produces “high morbidity and mortality rates, malnutrition, reduced life expectance, etc.” [29]. This is particularly prevalent amongst women living in the villages, because of the time spent weeding and their extensive contact with the soil.

There is, however, no uniform articulation of environment/health threats associated with *gandapani* and spinach farming in Karhera. As revealed by the in-depth interviews, and participatory mapping, not everyone is comfortable with this form of irrigation and with the consumption of produce grown in wastewater. As Umesh says “the spinach grown in *gandapani* is not healthy. The root absorbs the dirty water and the polluting agents. These agents then enter into the plant. So when we eat that it enters the human body and causes disease”. Amber Singh, who cultivates a variety of vegetables, avoids buying vegetables from the market as he cannot be sure about the water used to irrigate the vegetables. As he and his family are still able to irrigate their large landholdings with “clean water”, and they consume only this produce. Similarly, Bina, only eats spinach grown in “clean water”. She says that the wastewater used for irrigation contains latrine and toilet waste from all Karhera’s households: “Everybody has put pipes and there are neither ditches nor tanks. And the (toilet) waste goes directly to the drain. And so their crop grows faster. … the impact on health is apparent. We never eat that spinach”. Bina’s mother-in-law added that, recently, her son “had got *dhaniya* [coriander] and it looked dirty… When I picked up, I saw it had feces on it”. This was taken as a clear indication of health-harming contamination. These residents, who do articulate concerns with *gandapani*, have developed strategies to protect themselves from potential contamination. For example, because Kasturi Devi develops allergies when she is exposed to the wastewater, she wears shoes to protect her feet when working in the fields and, immediately after leaving the fields, washes her hands with Dettol. However, despite perceiving health risks related to *gandapani* spinach consumption, these residents’ concerns did not inspire collective mobilization to challenge the practice (discussed further below).

Some people believed, however, that the spinach or exposure to *gandapani* was not unhealthy and articulated this in interviews. Jeevan Lal, for example, argued “There is no disease here due to spinach cultivation”, and others agree that the water they use for irrigation is “just household water”. As such, and as Santosh explained, there is no harm in using *gandapani* for irrigation and consuming the spinach. Dhanush similarly points out that the “*gandapani* remains in the roots of the spinach”, and when the spinach is grown, the roots are thrown away. As a result, “there is no effect on health. And [this is evident because] for the past 16–17 years, sewage water is used to grow spinach [and no-one has become ill]”. These inhabitants consume this spinach and do not articulate any concerns about possible environment/health threats.

Air pollution, like water pollution, is highly visible in Karhera. During our first community meeting with village elders, they pointed to the black smoke emitted from one of the nearby factories. Residents complained that washing hanging on the line became contaminated with black soot and questioned whether this soot was also entering their lungs and causing harm. According to the villagers, air pollution was the reason behind the increasing cases of non-communicable diseases. They drew a direct correlation between the health of Karhera’s residents and the proximity of the factories, arguing that the factories caused tremendous harm. Tezpal Singh, for example, suggested that the toxins in the air could be causing cancer in the village. During a community mapping exercise, the men said:

Diabetes, cancer, high [blood] pressure can be seen more [frequently]. There is a factory at the vicinity of this village [referring to a dye factory which colors jeans and/or a rubber factory which burns rubber]. The smoke from this factory spreads into the village.

The polluted air from the industries is also seen to affect crops. During the same community mapping exercise, the men directly associated polluted air with a plant disease called *Chandi* (lit. silver) which destroyed crops. The glittering coating and fungus was most prevalent each year during Diwali, and residents linked this to the additional contaminants in the air, caused by factory emissions combining with Diwali pollution.

Others commented that the factories exerted a tremendous negative impact on the health and agriculture of the village. Umesh said “we can see black marks on the leaves of the plants. The leaves of the plants get covered with the tiny black dust that comes in the air from the factory”. Rajni argued that, sometimes, the spinach leaves in her fields shrink and dry due to this polluted air; at other times the leaves are infected by a fungus and her crop spoils.

## 4. Discussion: Peri-Urban Living

Environmental justice movements have worked with communities to challenge environmental pollution while simultaneously addressing health inequities [12,13,14]. In peri-urban areas, environmental justice issues are often particularly stark. While all peri-urban residents may experience air and water pollution, the poor are disproportionately affected in that they also lack decent sanitation and access to medical services, while working in unregulated conditions and with contaminated soil and crops [24,33], and may be particularly dependent on natural resources for their livelihoods [21,22]. Furthermore, they have far less ability to control their exposures and less choice. They cannot escape the unsavory water by purchasing expensive drinking water. Their constant and extensive exposure to a wide range of pollutants threatens their livelihoods and health. As Douglas argues, “there is a critical peri-urban human ecology where healthy crop plants and healthy human life go hand in hand” [24].

In other peri-urban areas of India, there has been considerable concern about vegetable production and the exposure to toxins and pollutants. Water and air pollution have been the subject of numerous newspaper articles and have, on occasion, resulted in civil society protests against polluting industries. In Karhera, there have only been a few isolated attempts to address environmental pollution despite official recognition of the contaminated environment [26]. Some of Karhera’s residents had complained about a particular factory to the police station. But, nothing was done. Efforts such as these underline the lack of collective action in Karhera. There are no community NGOs addressing pollution, there are no local attempts to treat wastewater before irrigating, there are no Karhera activists and no local political leaders articulating environmental concerns (discussed further below). The reasons for this stem, in part, from the diverse views on whether spinach grown in *gandapani* is damaging to health and in part from the heterogeneous nature of the community, the mutual interdependencies between these residents and the diverse ways in which different members of the community benefit (or lose out) from urbanization.

Conventional literature focuses on how low-income, marginalized racial or ethnic communities tend to experience much higher levels of exposure to toxins [34,35,36,37]. These differential levels of risk mean that low-income communities are often far more aware of the pollution and toxins than middle-class residents who are better able to control their environments through mitigating measures [38]. Few studies examine contexts where the people living in low-income settlements do not recognize the health challenges associated with toxins or pollutants (but see [37]) or contexts where poor, marginalized communities and middle-class residents live cheek-by-jowl and where recognition of hazards does not follow socio-economic divisions. However, in Karhera exposure to pollution and recognition of risk cannot be disaggregated by class or identity. As revealed by surveys, interviews, and participatory mapping, here almost all farmers engaging in agriculture are using wastewater. This includes men, women, upper-caste, lower-caste, migrants, and original inhabitants. Half of the original upper-caste, land-owning farmers interviewed were happy to eat spinach grown in *gandapani*, while the other half were not. But all of the non-landed, whether upper-caste or Dalit, migrant or original inhabitants, ate the spinach they produced. In this group, most people did not acknowledge any potential health threats. However, the poorest of the upper-caste original inhabitants who no longer own land, ate this spinach while articulating concerns about *gandapani*. We found no clear distinctions between men’s views and those of women. This lack of clear divisions reflects the heterogeneous nature of agriculturalists in Karhera. Here farming is undertaken by nearly half Karhera’s residents: original inhabitants and migrants (both long-term and recently arrived), people who have large and small land holdings, people who farm animals (buffalo and pigs), and people who farm crops, upper and lower-caste residents and migrants, hired workers, farmers who rent land, share-cropping farmers and land-owners tending their own lands. Maintaining a livelihood through agriculture in Karhera requires constant interactions and mutual dependency across social divides of class and identity. Land-owners may depend on rents earned from leasing arable land or cultivation of their own plots. Some poorer residents depend on opportunities to work in others’ fields, while others are engaged in purchasing and selling of produce in the markets. Ultimately, the large numbers of people directly or indirectly dependent on agriculture across the social spectrum is an influencing factor in continued marginal concern over health risks associated with *gandapani* cultivation and the consumption of produce grown under this method.

Mutual dependencies and the benefits of spinach production with *gandapani* water explain why mixed interpretations about the health/environment threats exist. Several studies have explored peri-urban communities’ needs and demands around water in India [3,26,39]. Mehta and colleagues argue that, in India, few peri-urban residents believe that there is any value in making demands on the state, rather “both the rich and poor opt out completely of the formal system and need to fend for themselves” [33]. Mehta et al.’s research, looking into water pollution and mobilization in India and Bolivia, they found that, “when pushed”, some Indian peri-urban residents said they would partake in collective action if organized by others, yet many others, such as migrant laborers, did not have formal residential status, felt more vulnerable and were unable to operate as “rights-bearing citizens” who could make sustainability and environmental justice demands on the state [33]. Instead of collective action and protest, India’s peri-urban residents have devised their own, informal strategies to ensure access to water [26,33,40]. However, in Karhera there are other well-known and recognized forms of pollution. Why have these too, for the most part, been accepted and why has there been no collective action to address these more obvious forms of pollution? The explanations lie partly in the nature of Karhera and its peri-urban location; partly in the actions of government authorities, and partly in the way pollution is discussed in the Indian media. An appreciation of the context in which people live adds a crucial dimension to local perspectives on, and apparent acceptance of, pollution. As Corburn and Karanja [7] argue, drawing on an African example, understanding the complexity of informal settlements and the diverse determinants of health, require a relational place-based approach which focuses on the ways in which context defines and shapes peri-urban residents’ perspectives and, in turn, informs policy.

### 4.1. Advantages and Disadvantages of Peri-Urban Living

Living in peri-urban Karhera has both advantages and disadvantages for all Karhera’s residents. This ambivalence is, as shown below, most clearly evident in *gandapani* spinach production.

Karhera’s upper-caste land-owning residents have retained the rhythms of rural life, their socio-cultural moorings, and ties to land. They are able to generate a livelihood from agriculture and have access to new urban markets for this produce. In some instances, spinach farming has been so lucrative that large land-owning original inhabitants have given up regular employment to focus on their farming. One such example is Bablu, who hires laborers to work in his fields and whose income today is more than he earned in his private job. Other advantages include, rather ironically, the fact that Karhera itself is rapidly urbanizing. Many upper-caste women prefer the *pucca* houses, concrete roads, electricity, and piped water. The submersibles ease women’s domestic labor and *gandapani* means they do not need to invest labor in the irrigation of their spinach fields. The village, with fewer buffalo and cows, is perceived to be cleaner. The value of land in and around Karhera has massively increased and has facilitated new, lucrative forms of income, including renting accommodation to migrants, selling land at increased rates to property speculators, and white collar employment for literate upper-caste members. These additional urban-informed incomes have, as Malin and DeMaster [22] point out in their analysis of environmental injustice caused by hydraulic fracturing on Pennsylvanian farms in the USA, supplemented often-times marginal and insecure agricultural practices, but have long-term consequences in terms of environmental inequality. They term this a “devil’s bargain” and in Karhera this takes the form of farmers’ dependence on both agricultural production and on urban- and industrially-influenced economic activities, which in combination leads to incremental environmental degradation while simultaneously shoring-up agricultural production.

The upper-caste has experienced modern lifestyles, education, and a shift from manual labor, yet many are disillusioned. They are acutely aware of their loss of land. They also repeatedly stress their failure to influence government officials who do not come to the village and do not listen to them. This leads to a sense of disempowerment. They have also lost their sense of control over the lower-caste residents and their collective sense of being “owners of Karhera village”. Although materially they survive relatively well on a combination of agriculture, rental agreements, and white collar jobs, unlike farming, this does not provide a sense of being their own masters. Karhera’s upper-caste residents also experience a lack of political clout. Previously, as large land-holders in an agricultural village, they would have been the political elite. They would have constituted the *panchayat* and engaged with members of local government, particularly in the form of the Department of Agriculture. However, as Karhera is now an urban ward, they no longer have access to these forms of rural governance. In addition, as the Department of Agriculture is concerned with cereals and grains, rather than vegetable farming, few political connections remain.

Conditions have also improved for lower-caste inhabitants of Karhera. Many of the original *Dalit* inhabitants no longer work in agriculture. Instead, they are in employment in general stores, as petrol pump attendants, car mechanics, drivers, painters etc. Some of these residents commute to Delhi or Ghaziabad daily, working as laborers or daily wage earners. A very small proportion of original *Dalit* inhabitants are employed as civil servants, teachers, or in the police force. The increasing urbanization of Karhera has also meant that *Dalit* women, now able to travel beyond the village boundaries, are finding work as domestic staff or as security guards in malls. Because original *Dalit* families never owned agricultural land and never kept buffalo, they had fewer opportunities to convert agricultural buildings into leased accommodation. Nonetheless, some families have been able to rent out one or two rooms in their homes. In some ways, *Dalit* villagers’ social standing has improved: in the past, the *Dalit* settlement or *basti,* located on the edge of the village, was also the dumping ground for animal carcasses and *Dalits* performed caste labor (cleaning the village, working on upper-caste fields, collecting firewood). The inflow of migrants and the rent economy has reduced these spatial and social caste distinctions. Explicit discrimination along the lines of caste is no longer a common feature in Karhera. Correspondingly, offensive language and caste-related slurs, have reduced. As is evident above, the opportunities created by *gandapani* and spinach have been particularly beneficial for Karhera’s *Dalit* farmers. Recall the example of Lukshmi, a single woman who transitioned from wage labor to being a farmer in her own right and hires land on which to produce spinach. Some *Dalit* men, like Shiv Kumar (described above), have been able to use it to set up their own businesses buying spinach from farmers and selling it in the market and, in this way, avoiding factory labor. A few *Dalit* families still practice pig husbandry (reared in their homes) to supplement their income.

Notwithstanding these improvements, Karhera’s original *Dalit* villagers also experience a sense of disempowerment. Not only are they being replaced by *Dalit* migrant laborers, but they remain at the bottom of the social ladder and its power hierarchy. The poorest of the original *Dalits*, people such as Manjari (see above), continue to work as laborers on other people’s fields. Some caste discrimination still remains: as one *Dalit* woman complained: “It was just the same, as it was in the past”. For the *Dalits,* as for the upper-caste, life in Karhera is ambivalent, with both advantages and disadvantages, and they too have accepted the trade-offs. Some *Dalits* have, however, no option but to engage in *gandapani* spinach production. They neither own large areas of land nor have the financial resources to invest in the technology required for “clean water” irrigation. For them, the only farming option is spinach cultivation using domestic wastewater. Their experience of disease and health has not deteriorated either, as they have always done manual labor and always been exposed to domestic waste. Other lower-caste studies have also found that people trade off health for social and economic improvements [20,21,41,42].

Migrants too find the experience of living in Karhera to be one of gains and losses. They have come to Karhera because it offers cheap housing, provides access to education for children, and to jobs in the nearby factories. These conditions far exceed their rural opportunities. For example, Durga and her husband came because they could “hardly manage” their livelihoods in Bihar. Reflecting on their move, she says:

Where would we get money, how would we look after our children? Our land in Bihar is barren, how could we do cultivation? Simple sewing does not give you a yield and good harvest. You need water and fertilizer to get a good yield. At least here my husband can work in the factory. I work as an agricultural laborer on the land of the villagers.

Similarly, Srichand left Meerut 20 years ago in search of work. Initially he got work in a dye factory in Karhera. He was subsequently diagnosed with tuberculosis (after about 5 years of work). When he recovered, he chose not to return to the factory and started selling spinach instead. As he was able to generate more than his wages, he has continued selling spinach. For many of the migrants, the work that they get in the spinach fields is better than factory work. As Durga’s comment suggests, Karhera offers a degree of economic and food security. For these reasons, few migrants complain about the use of *gandapani* in spinach production. Yet, despite the economic advantages of living in Karhera, the migrants complain of the arrogance and hostility of the upper-caste original inhabitants, and are acutely aware of their lack of wealth. Social tensions are palpable within Karhera village. Even though some migrants have lived in Karhera for more than 40 years and have bought their own homes, they are still referred to as outsiders. In addition, as industrial employers prefer migrant laborers who have less opportunity to unionize or demand better conditions, they are blamed for taking factory jobs away from original inhabitants. These jobs are seen to benefit only the migrants and not the villagers.

The janus-faced advantages and disadvantages of living in Karhera are symbolized in *gandapani* spinach production—which is productive and financially lucrative, but potentially damaging to health. Some upper-caste residents accept this trade off and therefore find no reason to complain, others have not accepted the trade off and thereby are ready to “see” the negatives as well. It is those upper-caste residents who have benefitted the least—the poorest of the village elite—who were most articulate about potential environment/health spinach threats. They, like other upper-caste villagers were aware of the media (see below), and of the villagers’ political marginalization, but, unlike other *Rajputs*, they did not have “clean water” fields, or spinach from these fields, to consume. They thus felt their deprivation the most.

### 4.2. Precarity and Lack of Community in Peri-Urban Karhera

While such ambiguities exist for spinach farming, it is clear that industrial air and water pollution—and the potential for ill-health—is more widely accepted. However, there is still no collective action around this. In part, this is because of the interdependencies in Karhera: the upper-caste farmers and other upper-caste residents of the village rely on the migrants to lease property; the *Dalits* also rely on the migrants for rental gains, and many of them generate an additional income through the industrial economy in the form of new and additional markets and trades. Migrants also need the original residents for both employment and accommodation. Yet, despite these interdependencies, there is no sense of community. As one original *Dalit* woman explained, “there is no unity at all”. Instead of a strong sense of community, our research gives a sense of people marking time in Karhera and life, as all these residents experience it today, is precarious. There are insecurities associated with the decreasing availability of water, the decline in arable land as more and more housing is erected, and, as middle-class expansion continues, many of Karhera’s residents fear that there will ultimately many be no place for them. Already they are absent from both government political processes and corresponding media reporting.

The media review makes it clear that, over the past 10 years, there have been frequent articles about pollution in peri-urban India, which predominantly address new middle-class concerns. Very few articles have examined the intersection between pollution, environmental degradation, and health, and seldom focus specifically and exclusively on Ghaziabad, reporting instead on either several areas or the broader Trans-Hindon or Delhi-National Capital Region areas (as the Hindon River divides Ghaziabad city and its peri-urban peripheries with the area to the west known as the Trans-Hindon and the East as Cis-Hindon). Only six articles reporting on the Trans-Hindon have linked cancer to the industrial discharge into the main rivers, including the Hindon. One article suggests that a Trans-Hindon village experiences high incidence of cancer and bone deformities resulting from the elevated levels of toxic metals in the rivers [43]. Another article points to the untreated industrial effluents and untreated or partially treated sewage in the Hindon River, and connect this to the unsafe levels of chromium, arsenic, and fluoride in groundwater [44]. Yet another reports on air pollution in the Delhi-NCR region, highlighting Ghaziabad as having rising SO_2_ and CO_2_ levels, and links these to different kinds of cancers, Tuberculosis, high blood pressure, kidney failure, and heart failure [45].

Residents of Karhera—particularly upper-caste landholders have read these media articles, as indeed they told us during interviews. As their above quotes show, they too link pollution to cancer. This led us to investigate actual cases of cancer in the village. The household survey and all in-depth interviews enquired about cancer diagnosis in the households. Only one *Dalit* household reported a current case of cancer, which was not explicitly linked to any form of pollution. This failure to demonstrate a significant burden of disease goes partway to explaining why residents believe that pollution is causing them harm, but have not done anything about it. It is, in keeping with recent literature of other cases of environmental pollution, a context in which the damage to the environment has been undramatic and gradual [20], and in which evaluating the extent of the risk, pinpointing responsibility and allocating blame remains unclear, difficult, and ambiguous [18,19]. This ambiguity in identifying who to target is also a consequence of the way political engagement in Karhera has waned over the years. Former rural local governance, such as the *Panchayat,* no longer exists and agricultural government officials are no longer interested in Karhera. Current government institutions which do cover Karhera are complicated and remote. Randhawa’s and Marshall’s examination of government policy and water management plans in peri-urban Ghaziabad shows the complexity of local government (three ministries are involved, two center-level subsidiary bodies and four departments) and argue that this creates a context in which national level policy makers are able to “exclude themselves from the larger context of the problem and to represent the government’s view”, emphasizing technical solutions and scientific expertise (pumping stations, sewerage treatment plants) to solve problems in the future [26]. It also marginalizes junior staff who interacted much more closely with peri-urban residents and were thus more aware of the problems on the ground, yet were unable to address the problems or persuade their seniors to act or—where there were official plans already in place—to release funding to enable local officials to address particular issues. Furthermore, the expert and technical nature of the policy process, and of the policies, operated to exclude other local forms of knowledge, despite the fact that India has encouraged participatory processes in government policy [26,46]. Local opportunities for people’s participation were thus unsympathetic to the grievances of the disempowered peri-urban residents, structured to facilitate the participation of middle-class urbanites, and, in any event, often unknown to poorer residents. Where Karhera’s residents do have an opportunity to participate in governance processes, such as through an elected municipal councilor of the Urban Local Body (ULB), these posts are not particularly powerful, are not integrated into all the different structures, and are highly dependent on the person elected. Randhawa and Marshall show that some councilors play an active role, while others only intervene when people’s access to resources are directly threatened. Ironically, in the case of Karhera, the current councilor is inactive and a former councilor has been involved in the development of urban facilities from which Karhera’s residents are excluded.

## 5. Conclusions

Veena Das has pointed to the “difficulty of theorizing the kind of suffering that is ordinary, not dramatic enough to compel attention” [47]. Illness can be normalized, and interpreted as the kinds of things that happen to bodies. Pollution can similarly be normalized, does not always lead to collective action and may not be facilitated through participatory processes [18,20]. Rather, collective action requires a recasting of illness or disease, resources, and a degree of social trust both in the community and in the state. As Kasperson and Kasperson argue, participatory engagement is a learned skill built up through years of interaction with political processes and government officials. “Left out” from the vision of participatory theory and collective action “are those who do not yet know that their interests are at stake, whose interests are diffuse or associated broadly with citizenship, who lack the skills and resources to compete, or who have simply lost confidence in the political process” [17]. The potential for using citizen science in contexts such as these, where residents do not represent a cohesive community and where the peri-urban economy has tied local residents into relationships of mutual dependency which inhibits political mobilization, despite some residents’ concerns about the health risks, remains to be explored in future research initiatives.

Pollution and contamination is complicated. In Karhera, pollution is obvious at one level (the “black river”) and hidden at another (the contaminants in “clean water” or in *gandapani*), and there are few direct sensory responses or collective biomedical consequences. As such, considerable uncertainty often exists about whether pollution is harmful or not, or how much exposure is safe. Yet the identification of hazards is not just about the science of pollution. As Kasperson and Kasperson argue, oftentimes societies do not recognize and acknowledge pollution, waste, and other industrial hazards, in part because of the nature of the hazard (uncertainty about the science, lack of sensory experience or high disease burdens), and in part because of the nature of the society (because the pollution serves other purposes in society). This is even more pertinent in peri-urban contexts, where a relational approach and a focus on place deepens this analysis. As Corburn and Karanja [7] have argued, the nature of the place itself shapes the possibilities for meaning-making in for health. In India, the peri-urban space is targeted for urban development and a means of attracting international firms and industry [48,49] and governance excludes those who are not a part of this urban vision. In contexts such as these, dependency on natural resources in combination with other urban- and industrially-derived incomes can “elide other critical social and environmental concerns” [22], leading to a “devil’s bargain” in which environmental degradation and health threats are accepted as part of a livelihood strategy. Karhera is just one small peri-urban village, caught up in and physically manifesting, broader processes of urbanization and globalization. But, it is a village in which the combination of a highly heterogeneous population, socio-economic tensions, and interdependencies between residents, and a lack of representation in the media, and political marginalization circumscribe residents’ ability to engage in collective action. Collective action for environmental justice in peri-urban places may, as a result of this combination of factors, be far from the development agenda.
